# Balancing automation and expertise: real-world evaluation of AI-assisted whole-exome sequencing in lupus-causing genes

**DOI:** 10.1136/lupus-2026-002049

**Published:** 2026-09-30

**Authors:** Anastasia-Vasiliki Madenidou, Gillian I Rice, Terence Garner, Sarah Dyball, Ben Parker, Alice Chieng, Adam Stevens, Ian N Bruce, Tracy A Briggs

**Affiliations:** 1 Centre for Musculoskeletal Research, The University of Manchester, Manchester, UK; 2 Kellgren Centre for Rheumatology, Manchester Royal Infirmary, Manchester University NHS Foundation Trust, Manchester, UK; 3 Division of Evolution, Infection and Genomic Sciences, The University of Manchester, Manchester, UK; 4 Manchester Centre for Genomic Medicine, Saint Mary’s Hospital, Manchester, UK; 5 Division of Developmental Biology and Medicine, The University of Manchester, Manchester, UK; 6 Department of Rheumatology, Royal Manchester Children’s Hospital, Manchester, UK

**Keywords:** Lupus Erythematosus, Systemic, Autoimmune Diseases, Autoimmunity

## Abstract

**Objective:**

Whole-exome sequencing (WES) is increasingly used to investigate patients with suspected monogenic forms of systemic lupus erythematosus (SLE) and other systemic autoimmune rheumatic diseases (SARDs). With the increasing use of artificial intelligence (AI)-assisted variant interpretation tools, there is a need to evaluate their real-world utility. In a SARD cohort, we compared the performance of AI-assisted and expert-guided WES analysis strategies in identifying disease-relevant variants within lupus-causing genes.

**Methods:**

We analysed WES data from 120 SARD patients using the same platform, Emedgene. For the AI-assisted analysis, a fully automated phenotype-based variant analysis produced a list of prioritised potentially disease-causing variants for each case, labelled as ‘most likely’. For the expert-guided approach, expert-informed filtering criteria were applied. Both the ‘most likely’ AI-assisted and the expert-informed filtered variants then underwent further manual analysis.

**Results:**

Before manual analysis, a total of 2906 variants potentially likely to be causative were identified with the Emedgene AI tool (mean 20.8 variants per case), approximately three times fewer than the 8391 variants identified by the expert-guided method (mean 59.9 per case). A comparable number of variants (78 vs 56) in lupus-causing genes were identified at the end of the manual analysis with both methods, including two monogenic cases *PEPD* (c.819-1G>A protein change, homozygous) and *CYBB* (c.1314+1G>A protein change, hemizygous).

**Conclusions:**

AI-assisted WES analysis substantially reduced the number of variants requiring manual review without missing the two monogenic cases. These findings support the integration of AI-assisted pipelines into genomic workflows for SLE and other SARDs which has the potential to improve efficiency and scalability of WES.

**WHAT IS ALREADY KNOWN ON THIS TOPIC**

**WHAT THIS STUDY ADDS**

- Demonstration that AI-assisted analysis substantially reduces the number of variants in lupus-causing genes requiring manual review while not missing two monogenic cases.
- Provision of a pragmatic, clinically applicable framework for integrating AI into autoimmune genomics workflows.

**HOW THIS STUDY MIGHT AFFECT RESEARCH, PRACTICE OR POLICY**

- The efficiency of AI could enable the scalable implementation of WES in lupus and other SARDs, facilitating improved understanding of their genetic architecture.

## Introduction

 Systemic autoimmune rheumatic diseases (SARDs) are chronic autoimmune diseases with a significant genetic contribution to their aetiology. While the genetic contribution to SARDs is usually attributed to an aggregation of weak-acting, common variants, many monogenic causes of SARDs and especially of systemic lupus erythematosus (SLE) have now been identified.^1^ As our understanding of the genetic basis of immune dysregulation expands, whole-exome sequencing (WES) is increasingly used to evaluate patients with suspected monogenic autoimmunity or to explore complex disease architecture.^2^

Until recently, the clinical interpretation of genetic variants in relation to a patient’s phenotype was a largely manual, labour-intensive process that relied heavily on expert input.^3^ Artificial intelligence (AI) tools are now increasingly used, enabling researchers to analyse vast amounts of genomic data more efficiently.^3^ These tools use integrated databases and filtering pipelines to prioritise rare, potentially pathogenic variants. However, their performance in autoimmune phenotypes is uncertain.

In this study, we compared two WES analysis strategies in a SARD cohort by focusing on known variants in lupus-causing genes: an AI-assisted pipeline and an expert-guided approach using the same platform called Emedgene.^4^

## Material and methods

WES, using the Illumina NovaSeq6000, was undertaken in 120 patients and 20 healthy controls (HCs) in the Lupus Extended Autoimmune Phenotype (LEAP) cohort at the Genomic Technologies Core Facility, the University of Manchester (table 1). The LEAP cohort is a UK prospective multicentre study of adult and paediatric patients with a diagnosed SARD-SLE, mixed connective tissue disease, systemic sclerosis, idiopathic inflammatory myopathy, Sjögren’s disease (SjD) and undifferentiated connective tissue disease (UCTD) (ethical approval: 13/NW/0564).

**Table 1:** Demographic characteristics of SARD patients undergoing WES

	Patients (n=120)	Healthy controls (=20)
Age at diagnosis, years	35 (28)	NA
Diagnosis in childhood	22 (18.3%)	NA
Age at WES (years)	49.5 (26.3)	37 (19.5)
Sex (female)	94 (78.3%)	16 (80%)
Ethnicity		
White	83 (69.2%)	17 (85%)
Asian	16 (13.3%)	1 (5%)
Black	18 (15%)	1 (5%)
Other	3 (2.5%)	1 (5%)
Disease		
SLE	42 (35.0%)	NA
SjD	20 (16.7%)	NA
MCTD	20 (16.7%)	NA
UCTD	19 (15.8%)	NA
SSc	12 (10.0%)	NA
IIM	7 (5.8%)	NA

Age is median (IQR).

IIM, idiopathic inflammatory myopathy; MCTD, mixed connective tissue disease; SARD, systemic autoimmune rheumatic disease; SjD, Sjogren’s disease; SLE, systemic lupus erythematosus; SSc, systemic sclerosis; UCTD, undifferentiated connective tissue disease; WES, whole exome sequencing.

FASTQ files and phenotypes mapped to Human Phenotype Ontology (HPO) terms^5^ were submitted to Emedgene.^4^ The Emedgene platform performs automated phenotype-driven variant prioritisation using AI, integrating genomic data with patient phenotypic features encoded through HPO terms. The platform generates a list of prioritised variants for each case that are more likely to be causative, labelled as “most likely’.^4^ Additional variants that are highly scored and worth considering are labelled as ‘candidates’. Although the algorithms underlying the prioritisation process are not publicly available, the platform provides transparent evidence supporting each prioritised variant.^6^ This includes links to relevant scientific literature, known disease-gene associations, population frequency data, inheritance patterns, pathogenicity predictions and information from established genomic databases. The ‘most likely’ variants, based on all available data from Emedgene, were extracted into a spreadsheet for further analysis. The categories of data available for each case are included in the online supplemental table 1.

For the expert-guided approach, the Emedgene platform was used for the initial part of the analysis as it allows the application of filters. Variants were selected: (a) which were pathogenic, likely pathogenic and of uncertain significance according to the American College of Medical Genetics and Genomics^7^ classification and (b) with specific main effect (splice acceptor variant, splice donor variant, stop gained, frameshift variant, stop lost, start lost, inframe insertion, inframe deletion, protein altering variant, missense variant). Then, all variants with all available data were extracted^8^ (online supplemental table 1).

For both analytical approaches, variants were next manually filtered to include only those located in lupus-causing genes (online supplemental table 2) and previously reported on the comprehensive public archive of human genetic variants, ClinVar^9^ (labelled as ‘known variants’ in the Emedgene-derived spreadsheet; online supplemental table 1). Subsequently, variants with a gnomAD allele frequency^10^ exceeding 0.003159 were excluded; this threshold was chosen to enrich for rare variants while avoiding overly restrictive filtering. Variants with allele frequencies below 0.001^11^ are commonly considered ultra-rare, whereas thresholds below 0.01^12^ are frequently used to define rare variants in genomic studies. To maximise inclusivity while maintaining a focus on rare variation, we selected the highest allele frequency within this range present in our dataset (0.003159) as the filtering threshold.

The final step for both methods included the evaluation of each variant for literature-based association with autoimmunity based on information available on ClinVar,^9^ MobiDetails^13^ and PubMed. Each variant was classified into one of the four categories: (1) variants with established association with autoimmunity and compatible inheritance, (2) variants with established association with autoimmunity but incomplete inheritance, (3) pathogenic or likely pathogenic variants on ClinVar but with no established association with autoimmunity and (4) variants of uncertain significance or classified as benign or likely benign or with conflicting classification in ClinVar. Incomplete inheritance refers to heterozygous variants in genes known to be disease-causing in the autosomal recessive or in females in the X-linked (hemizygous) state. A flowchart with the steps of variant curation for both methods is presented in figure 1.

**Figure 1:**
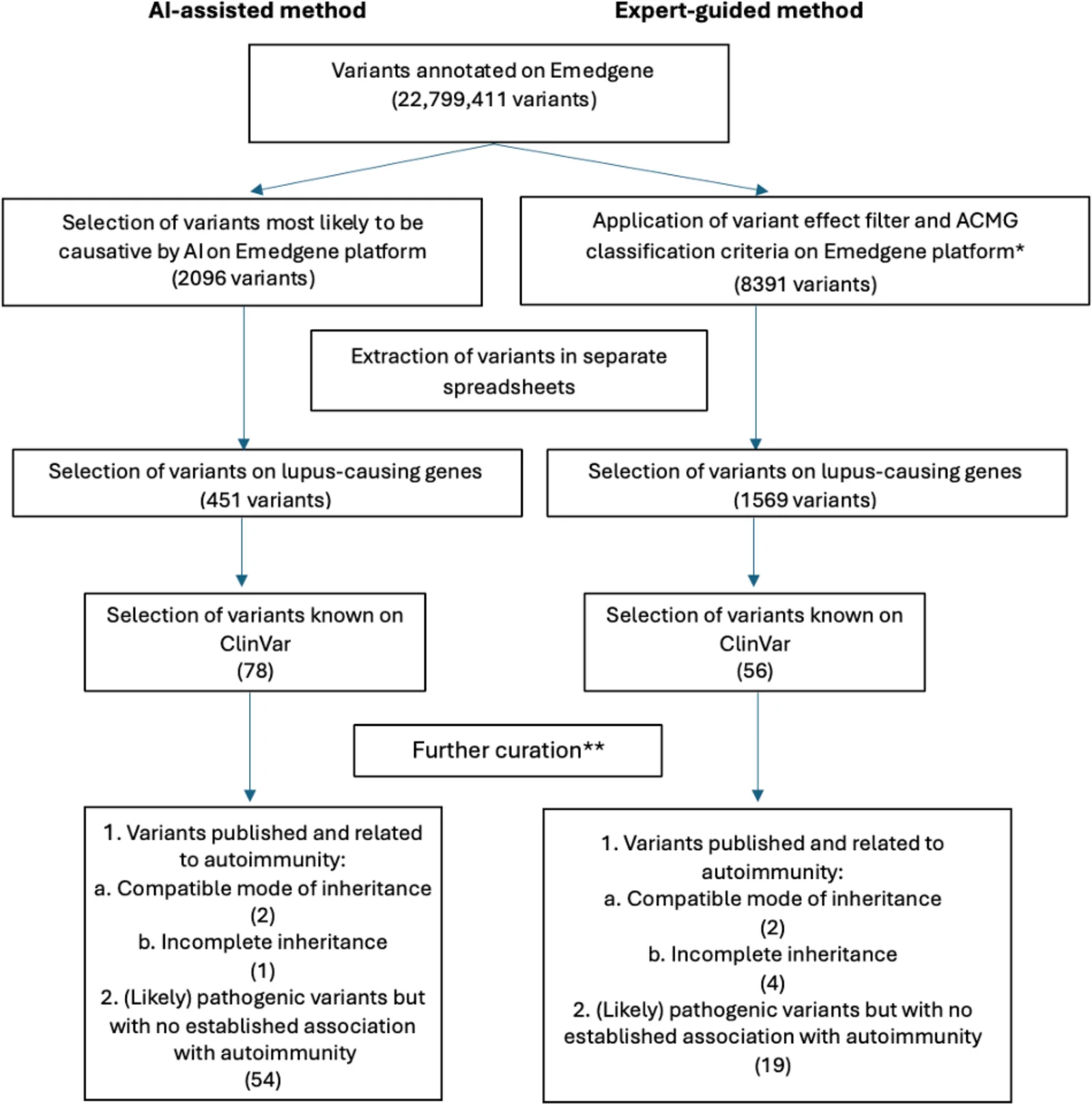
Flowchart comparing the variant curation process and the number of variants at each step using an AI- and expert-guided approach in a SARD cohort. *Variants were selected: (a) which were pathogenic, likely pathogenic and of uncertain significance (VUS) according to the ACMG classification and (b) with specific main effect (splice acceptor variant, splice donor variant, stop gained, frameshift variant, stop lost, start lost, inframe insertion, inframe deletion, protein altering variant, missense variant). **First step: variants were filtered out if gnomAD frequency was greater than 0.003159. Second step: variants were excluded when annotated in ClinVar as benign, likely benign, or of uncertain significance, when conflicting evidence regarding pathogenicity was present or when identified in individuals with a non-compatible phenotype. ACMG, American College of Medical Genetics and Genomics; AI, artificial intelligence; SARD, systemic autoimmune rheumatic disease.

## Results

The total number of identified variants was 22 799 411 (162 853 per case). A flowchart with the curated variants per step is presented in figure 1. Before the analysis outside the platform, a total of 2906 variants likely to be causative were identified with the Emedgene AI tool across all 140 cases (mean 20.8 variants per case) compared with 8391 variants (mean 59.93 variants per case) with the expert-guided method. The majority of variants with both methods were loss-of-function variants (frameshift, splice donor, splice acceptor, start lost, stop gained and stop lost variants): 1623 variants (77.4%) with the AI-assisted variant analysis and 8044 (95.9%) with the semi-automated approach. The breakdown of variants identified by each method and the overlap between them are presented in table 2. The common variants between the two methods were 1397 with 6994 having been uniquely identified with the expert-guided method and 1509 with the AI-assisted one.

**Table 2:** The variant types identified by the automated AI-assisted pipeline and the expert-guided approach, including both the shared variants and those uniquely detected by each method

Variant type	AI-assisted variant analysis	Expert-guided variant analysis	Common variants	Unique variants identified with the AI-assisted variant analysis	Unique variants identified with the expert-guided variant analysis
Loss-of-function variants (frameshift, splice donor, splice acceptor, start lost, stop gained, stop lost)	1623	8044	1327	296	6717
Transcript ablation/amplification	57	0	0	57	0
Inframe deletion/insertion	5	100	5	0	95
Missense	329	246	65	264	181
Coding sequence intron, intron, feature elongation, upstream, downstream	528	0	0	528	0
Protein altering	0	1	0	0	1
Splicing (not acceptor/donor)	338	0	0	338	0
Synonymous, 3’UTR, 5’UTR, non-coding transcript	26	0	0	26	0
Total	2906	8391	1397	1509	6994

AI, artifical intelligence.

The number of variants per patient was similar across diseases within each method, ranging from 19.76 to 26.57 with the AI-assisted approach and from 61.83 to 72.15 with the semi-automated variant analysis (table 3). In contrast, HCs showed a much lower variant burden compared with the average number of variants per patient across disease groups, only 7% as many variants (4.85 vs 69.22) as SARD patients with the expert-guided method and 66.3% (15 vs 22.62) with the AI-assisted approach.

**Table 3:** Number of variants per patient across disease groups and healthy controls identified by an AI-assisted pipeline and an expert-guided approach

	AI-assisted variant analysis	Expert-guided variant analysis
SLE	19.76	66.42
SSc	22	61.83
IIM	26.57	69.57
MCTD	21.7	69.45
UCTD	22.78	72.15
SjD	22.95	75.9
Healthy controls	15	4.85

IIM, idiopathic inflammatory myopathies; MCTD, mixed connective tissue disease; SjD, Sjogren’s disease; SLE, systemic lupus erythematosus; SSc, systemic sclerosis; UCTD, undifferentiated connective tissue disease.

With the AI-assisted method, variants in 50 known lupus-causing genes and 431 in other genes were identified, compared with 65 and 630 with the semi-automated method respectively.

After selecting variants in known lupus-causing genes only (figure 1), the number of variants dropped to 451 with the AI- and 1569 with the expert-guided method respectively, with a further decrease to 78 and 56 after excluding variants not previously reported on CliniVar.^9^

After incorporating literature-based evidence for variant–autoimmunity associations in the final selection step, the same two monogenic cases were retained. More specifically, a known pathogenic variant in *PEPD* (c.819-1G>A, homozygous),^14^ previously linked with monogenic SARDs, was detected in an SLE patient by both methods. A hemizygous pathogenic variant in *CYBB* (c. c.1314+1G>A), previously linked with the immunodeficiency X-linked chronic granulomatous disease (CGD),^15^ was identified in a male patient with UCTD by both analytical methods. *CYBB* mutations, but not this specific variant, have been reported in the context of SARDs.^16^ In keeping with the phenotype of CGD, the patient has a history of cervical granulomatous lymphadenitis confirmed by FNA but no recurrent infections. Interestingly, the same mutation was identified in three female patients, two with SLE and one with SjD, by the expert-guided method, suggesting that this variant could be implicated in the genetic aetiology of SARDs in female cases. Functional work is required to further investigate this. The two SLE cases have a history of lymphadenopathy. AI did not include these variants in the ‘most likely’ list, but they were in the ‘candidates’ list.

Using the expert-guided method, a pathogenic variant in *ADA2* (c.973-2A>G, heterozygous) was identified in a SjD patient. As with the *CYBB* mutation, the AI did not include this variant in the ‘most likely’ list, but it was included in the ‘candidates’ list. The clinical relevance of this variant is unclear, as it is a single heterozygous variant and the patient does not exhibit vasculitic symptoms associated with homozygosity for this variant.^17^

## Discussion

This is the first study comparing AI and expert-guided curation of variants in lupus-causing genes. Using the Emedgene platform and following a standardised approach, we demonstrated that both methods identified the same two monogenic cases.

The AI-assisted analysis initially identified three times fewer candidate variants than the expert-guided approach (2906 vs 8391), with 1397 variants shared between the two methods. This difference was maintained when restricting the analysis to lupus-causing genes, with the AI-assisted method identifying approximately 3.5 times fewer variants (451 vs 1569). Following analysis, the number of variants requiring literature-based interpretation was similar, 78 vs 56, respectively.

Notably, although the genes in our gene list are typically considered lupus-causing, the number of variants was comparable across SARDs, suggesting a shared genetic burden. As expected, the number of variants in HCs was smaller with both methods; however, the expert-guided approach showed a clearer separation between patients and HCs (7% as many variants in HCs as in SARD patients with the expert-guided method, compared with 66.3% using the AI-assisted approach).

The additional identification of a heterozygous *CYBB* variant in three female patients is noteworthy, as these variants could contribute to the clinical phenotype and could explain the heterogeneity and clinical complexity often observed in CTD patients. For example, two of the three heterozygous patients had a history of lymphadenopathy, which could be related to *CYBB*. Skewed X inactivation could contribute to disease in heterozygous females, and further functional work would be required to prove this.

The three heterozygous *CYBB* variants and one heterozygous *ADA2* variant were identified by the expert-guided approach but were not included in the AI-generated ‘most likely’ prioritised list. Importantly, all four variants were present within the AI-generated candidate list, indicating that the platform recognised their potential relevance but assigned them a lower prioritisation rank. These findings suggest that restricting analysis to the ‘most likely’ list alone may result in potentially relevant variants being overlooked in selected cases. Therefore, review of the candidate list may be warranted when there is a strong clinical suspicion of an underlying monogenic disease or when the initial prioritised results do not adequately explain the phenotype. While the AI-assisted approach substantially reduced the number of variants requiring review, these findings highlight the continued importance of expert oversight and clinical judgement in the interpretation of genomic data.

The study focused on known pathogenic and likely pathogenic variants in lupus-causing genes, not on novel disease-causing genes or variants. We restricted our analysis to these variants to enable a confident and controlled comparison of the two analytical methods. It is possible that additional genes or variants contributing to the development of SARDs are present in the WES data but were not captured by either method. However, investigating such findings is beyond the scope of this project. Further studies will be required to determine the utility of AI-assisted variant prioritisation for gene discovery applications.

It is important to note that although we refer to the two approaches as AI-assisted and expert-guided, both ultimately required expert input for variant interpretation. This highlights that while AI cannot fully replace manual variant curation, it can substantially reduce the number of variants to a manageable level for human review. Additionally, our findings cannot be directly extrapolated to other AI-based genomic tools, as this study was conducted using Emedgene. Nevertheless, the results highlight the potential role of AI in variant interpretation, and the systematic approach we used provides a framework that could be adapted to other AI platforms.

A limitation of our study is that the initial analysis for both analytical methods was conducted on the same platform, which could introduce bias across the methods, as Emedgene performed variant annotation. Nevertheless, using the same platform ensured that variants remained directly comparable. It should also be acknowledged that performing the expert-guided analysis within Emedgene likely underestimates the time and effort normally required for variant annotation and criteria application, meaning that the efficiency gains associated with AI-assisted variant prioritisation are likely greater in fully manual variant analysis. A further limitation is that our cohort may not have been predominantly monogenic in origin, in that we did not include, for example, families with multiple affected family members, thus reducing the likelihood of identifying many monogenic cases. At the same time, this reflects a real-world SARD cohort demonstrating that a small number of monogenic cases are encountered in routine clinical practice. The relatively small number of confirmed monogenic cases limited our ability to calculate rank-based performance metrics. Larger studies enriched for monogenic disease will be required to address this question.

Although formal time measurements and health-economic analyses were beyond the scope of this study, we provide approximate estimates of potential time and staff cost savings to contextualise the operational impact of AI-assisted variant prioritisation. The AI-assisted approach reduced the number of variants requiring manual review by approximately three times compared with the expert-guided approach within the same platform (2906 vs 8391 variants across the cohort). Assuming a conservative average review time of 5 min per variant, this would correspond to approximately 242 hours of analyst review for the AI-assisted workflow compared with 699 hours for the expert-guided workflow, representing an estimated saving of 457 analyst hours, equivalent to approximately 57 full working days based on an 8-hour working day. As this study was conducted in the UK, applying salary costs from Clinical Scientist/Bioinformatician posts (£47 810–£54 710/year),^18^ this would correspond to an estimated saving of approximately £11 000–£13 500 in staff time across the cohort. In a fully manual workflow outside an integrated annotation and prioritisation platform, the time required for variant annotation, filtering, phenotype matching and evidence review would be expected to be higher. Assuming 10–15 min per variant for fully manual curation of the 8391 expert-guided variants, the total review time would be approximately 1399–2098 analyst hours, corresponding to approximately £34 000–£40 000 to £51 000–£60 000 in staff time. These estimates are illustrative and do not include software licensing, infrastructure costs, institutional overheads or formal health-economic modelling.

Other studies outside rheumatology have also demonstrated the potential role of AI to expedite genome interpretation. Meng *et al* demonstrated that an AI-based model successfully prioritised pathogenic variants in the majority of confirmed monogenic cases.^19^ Similarly, De la Vega *et al* reported that an AI-driven genome interpretation tool accurately ranked causal genes in over 90% of cases with known Mendelian disorders.^3^

Our findings suggest that AI can effectively support variant curation; however, expert oversight remains essential to ensure accurate and clinically meaningful interpretation. By improving efficiency, AI has the potential to expand access to WES, thereby increasing the identification of monogenic diagnoses and helping to disentangle the contribution of rare variant burden in autoimmune genomics. We also outline a practical framework for integrating AI into the variant-curation workflow. Further work in targeted monogenic SARD cohorts or using additional AI-based genomic tools would help clarify the ultimate role of AI in this process.

## Supplementary material

10.1136/lupus-2026-002049 online supplemental file 1

## Data Availability

No data are available.
