# Supplementary material for: Balancing automation and expertise: real-world evaluation of AI-assisted whole-exome sequencing in lupus-causing genes
Source: Lupus Sci Med. 2026 Sep 30;13(2):e002049. doi: 10.1136/lupus-2026-002049 (PMC13630107; doi:10.1136/lupus-2026-002049)
Supplement: Supplementary file 1 [file lupus-13-2-s001.docx]

**Supplementary material**

**Table 1** Data categories extracted from the Emedgene platform

**Table 2** Lupus-causing genes

**Table 1** Data categories extracted from the Emedgene platform

| **Case id** | **Alternate Read** | **SpliceAI DS AG** | **Disease Inheritance Mode** | **Variant Inheritance** |
| --- | --- | --- | --- | --- |
| **LEAP ID** | **Proband Zygosity** | **SpliceAI DS AL** | **SIFT Pred** |  |
| **Phenotype** | **GnomAD AF** | **SpliceAI DS DG** | **LRT Pred** |  |
| **Chromosome** | **GnomAD AC** | **SpliceAI DS DL** | **MutationTaster Pred** |  |
| **Position** | **GnomAD SV AF** | **ExAC pLI** | **Polyphen2_HVAR Pred** |  |
| **REF** | **GnomAD SV AC** | **gnomAD pLI** | **Polyphen2_HDIV Pred** |  |
| **ALT** | **Max AF** | **Missense z-score** | **DANN Score** |  |
| **END** | **Pathogenicity** | **pRec** | **REVEL Score** |  |
| **Vartype** | **Coding Change** | **Known Variants** | **PrimateAI3D Score** |  |
| **Gene** | **Protein Change** | **Other Family Members** | **PrimateAI3D Prediction** |  |
| **Transcript** | **Homozygous** | **Allele Bias** | **Variant length (kbp)** |  |
| **Effect** | **Hemizygous** | **Reference** | **Phenomeld Score** |  |
| **Quality** | **Tag*** | **Evidence Text** | **AI Rank** |  |
| **Depth** | **Prediction** | **Diseases** | **Individual Genotype** |  |
| REF=reference allele, ALT=alternate allele, GnomAD=Genome Aggregation Database, AF=Allele Frequency, AC= Allele Count, SV= Structural Variant, DS= Delta Score, AG= Acceptor Gain, AL= Acceptor Loss, DG= Donor Gain, DL= Donor Loss, ExAC=Exome Aggregation Consortium, pLI=Probability of Loss-of-Function Intolerance, pRec= Probability of Recessive LoF Intolerance, SIFT = Sorting Intolerant From Tolerant, Pred= Prediction, LRT= Likelihood Ratio Test, DANN = Deleterious Annotation of genetic variants using Neural Networks, REVEL = Rare Exome Variant Ensemble Learner  *Most-likely or candidate options depending on Emedgene variant prioritisation | | | | |

**Table 2** Lupus-causing genes

| ACP5 | DOCK11 | NLRP3 | RNASEH2B |
| --- | --- | --- | --- |
| ADA2 | DOCK8 | NOD2 | RNASEH2C |
| ADAR | FAS | NRAS | RNF31 |
| AIM2 | FASLG | OAS1 | RNU7-1 |
| AIRE | FCGR2A | OGFRL1 | SAMHD1 |
| BACH2 | FCN3 | OTUD1 | SAT1 |
| BANK1 | GALR3 | OTULIN | SEC23B |
| BMP6 | GP6 | P2RY8 | SERPING1 |
| C1QA | GPR39 | PACSIN1 | SH2B3 |
| C1QB | HOXB1 | PADI4 | SH3BP2 |
| C1QC | IFIH1 | PDCD1 | SHOC2 |
| C1R | IKZF1 | PDE4A | SLAMF1 |
| C1S | IL17RA | PEPD | SLC29A3 |
| C2 | IL1RN | PIK3CD | SLC7A7 |
| C3 | IL2RB | PLCG2 | SOCS1 |
| C4A | IL36RN | PNP | SOS1 |
| C4B | IRF2BP2 | PRAMEF13 | STAT1 |
| C5 | IRF3 | PRF1 | STAT2 |
| C6 | IRF5 | PRKCD | STAT4 |
| C7 | IRF7 | PRKDC | STING1 |
| C8A | ISG15 | PRPF18 | TALDO1 |
| C8B | ITGAM | PSMA3 | TLR3 |
| C9 | JAK1 | PSMB4 | TLR5 |
| CARD14 | KRAS | PSMB8 | TLR7 |
| CGAS | LPIN2 | PSMB9 | TLR8 |
| CHMP6 | LRBA | PSTPIP1 | TLR9 |
| COPA | LSM11 | PTEN | TNFAIP3 |
| CR2 | MAN2B1 | PTPN11 | TNFRSF1A |
| CTLA4 | MAPK | PTPN2 | TNFSF13B |
| CXCL10 | MAVS | PTPN22 | TNFSF4 |
| CXCL9 | MEFV | RAF | TNIP1 |
| CXCR3 | MFGE8 | RAF1 | TRAF6 |
| CXCR5 | MIR146A | RAG1 | TREX1 |
| CYBB | MLPH | RAG2 | TRIM25 |
| DDX58 | MVK | RBCK1 | TRIM47 |
| DHX58 | MYD88 | RELA | TYK2 |
| DNASE1 | NCF1 | RIGI | UBA1 |
| DNASE1L3 | NLRC4 | RNASEH2A | UNC93B1 |
| DNASE2 | NLRP12 |  |  |
